# Prognostic role of the pretreatment systemic immune-inflammation index in patients with glioma: A meta-analysis

**DOI:** 10.3389/fneur.2023.1094364

**Published:** 2023-03-08

**Authors:** Sunhuan Zhang, Qunqin Ni

**Affiliations:** ^1^Clinical Laboratory, Huzhou Central Hospital, Affiliated Central Hospital of Huzhou University, Huzhou, Zhejiang, China; ^2^Clinical Laboratory, Traditional Chinese Medical Hospital of Huzhou Affiliated to Zhejiang Chinese Medical University, Huzhou, Zhejiang, China

**Keywords:** systemic immune-inflammation index, glioma, prognosis, meta-analysis, immune responses

## Abstract

**Background:**

The systemic immune-inflammation index (SII) has been recognized as the indicator that reflects the status of immune responses. The SII is related to the prognostic outcome of many malignancies, whereas its role in gliomas is controversial. For patients with glioma, we, therefore, conducted a meta-analysis to determine if the SII has a prognostic value.

**Methods:**

Studies relevant to this topic were searched from 16 October 2022 in several databases. In patients with glioma, the relation of the SII level with the patient prognosis was analyzed based on hazard ratios (HRs) as well as corresponding 95% confidence intervals (CIs). Moreover, subgroup analysis was conducted to examine a possible heterogeneity source.

**Results:**

There were eight articles involving 1,426 cases enrolled in the present meta-analysis. The increased SII level predicted the dismal overall survival (OS) (HR = 1.81, 95% CI = 1.55–2.12, *p* < 0.001) of glioma cases. Furthermore, an increased SII level also predicted the prognosis of progression-free survival (PFS) (HR = 1.87, 95% CI = 1.44–2.43, *p* < 0.001) in gliomas. An increased SII was significantly associated with a Ki-67 index of ≥30% (OR = 1.72, 95% CI = 1.10–2.69, *p* = 0.017). However, a high SII was not correlated with gender (OR = 1.05, 95% CI = 0.78–1.41, *p* = 0.734), KPS score (OR = 0.64, 95% CI = 0.17–2.37, *p* = 0.505), or symptom duration (OR 1.22, 95% CI 0.37–4.06, *p* = 0.745).

**Conclusion:**

There was a significant relation between an increased SII level with poor OS and the PFS of glioma cases. Moreover, patients with glioma with a high SII value have a positive relationship with a Ki-67 of ≥30%.

## Introduction

Primary brain tumors in adults are gliomas, which are the most common and deadliest type of tumors (1). Glioma accounts for 75% of all malignant adult primary brain tumors, and the 5-year survival rate of glioma is <10% (2). Glioblastoma (GBM) is a frequently seen brain cancer, accounting for 57% of all gliomas and 48% of all malignant CNS tumors (3). Among the standard medical treatments for GBM, resections are followed by external beam radiation and temozolomide (TMZ), followed by maintenance chemotherapy (4). In spite of these treatments, the prognosis of adult GBM is extremely dismal, its median survival time after diagnosis is <15 months, and its 5-year survival rate is as low as 5% (5, 6). The dismal prognosis of glioma is partially due to the lack of effective prognostic biomarkers (7). Some circulating biomarkers have been investigated to predict the prognosis of glioma; however, the limited specificity and sensitivity may compromise the clinical application (7). Therefore, to improve the survival of patients with glioma, it is urgently needed to identify cost-effective and reliable prognostic markers.

The systemic immune-inflammation index (SII) represents the plain prediction biomarker for inflammation (8). It was proposed in 2014 that the SII could be used for predicting the prognosis of hepatocellular carcinoma (HCC) cases undergoing surgery (9). The SII formula is as follows: SII = platelet count × neutrophil count/lymphocyte count. It is easy to obtain the SII value from a routine laboratory test in clinical settings, and the SII is cost-effective. In the past, SII has been demonstrated to have a significant prognostic effect in a wide variety of cancers in many studies, including gastric cancer (GC) (10), cervical cancer (CC) (11), renal cell carcinoma (RCC) (12), diffuse large B-cell lymphoma (DLBCL) (13), and melanoma (14). SII has also been investigated in numerous studies to be the prognosis prediction biomarker for glioma cases; however, results have been inconsistent (15–22). For example, some researchers reported elevated SII as the important biomarker to predict the prognosis of glioma cases (17, 19, 20), whereas some other scholars found no evident relation of SII with glioma prognosis (18). These conflicting results confuse the application of SII in the clinical management of patients with glioma. Therefore, for determining whether SII could be used to predict the glioma prognosis, we collected data from the most recent studies and carried out a meta-analysis.

## Materials and methods

### Study guideline

The present meta-analysis was performed following the instruction of Preferred Reporting Items for Systematic Reviews and Meta-Analyses (PRISMA).

### Search strategy

We searched Embase, PubMed, Cochrane Library, and Web of Science databases to identify related articles using the following keywords: (systemic immune-inflammation index OR SII) AND (glioma OR glioblastoma OR medulloblastoma OR oligodendroglioma OR astrocytoma OR brain tumor). In addition, English publications were considered to be qualified. The search duration was from inception to 16 October 2022. Reference lists from related articles were also manually checked for identifying possibly eligible articles.

### Inclusion and exclusion criteria

Studies conforming to the following criteria were enrolled: (i) the diagnosis of glioma was pathologically or histologically confirmed; (ii) the studies reported the relationship between the SII and survival in patients with glioma, regardless of whether the relationship is statistically significant; (iii) hazard ratios (HRs), as well as associated 95% confidence intervals (CIs), were available or calculable through given information; (iv) a SII threshold was used to classify cases as a high or low SII group; and (v) language of publication, i.e., English. The criteria for exclusion were as follows: (i) comments, reviews, conference abstracts, case reports, and letters; (ii) duplicate studies; (iii) non-human studies; and (iv) lack of sufficient data for analysis in studies.

### Data extraction and quality assessment

Qualified articles were included by two reviewers (SZ and QN), and information was collected independently. The disagreements were resolved through discussion and consensus. Data below were recorded, including first author, country, publication year, sample number, age, gender, study duration, pathology, study design, treatment method, follow-up, SII threshold, cutoff determination method, survival, type of survival analysis, HRs, and associated 95% CIs. Overall survival (OS) was the primary outcome, whereas progression-free survival (PFS) was the secondary outcome. Study quality was evaluated by the Newcastle–Ottawa Scale (NOS) (23). It covers three domains, namely, patient screening (0–4 points), research group comparability (0–2 points), and outcome evaluation (0–3 points), yielding a total score of 0–9 points. A NOS score of ≥6 was the criterion to select high-quality articles.

### Statistical analysis

Combined HRs together with associated 95% CIs of patients with glioma were computed for determining whether SII could be used to predict the prognosis of OS and PFS. Inter-study heterogeneity was assessed with Higgin's I^2^ statistics and Cochran's Q test. The random effects model was utilized to pool data in the case of an obvious heterogeneity (*p* < 0.10 or I^2^ > 50%); otherwise, the fixed-effects model was adopted. The potential heterogeneity source was analyzed by subgroup analysis according to a variety of factors. Moreover, this study adopted odds ratios (ORs) together with associated 95% CIs for analyzing the relation of the SII with patient clinicopathological features. By excluding any single study, sensitivity analyses were conducted to identify the source of heterogeneity and demonstrate the stability of the pooled results. Possible publication bias was analyzed using Begg's funnel plot and Egger's test. Stata software version 12.0 (Stata Corporation, College Station, TX, USA) was utilized for statistical analysis. A *p* < 0.05 was considered to be statistically significant.

### Ethics statement

The analysis was performed based on previously published literature and did not include personal data, so patient consent and ethical approval were waived for this study.

## Results

### Literature search process

**Figure 1:**
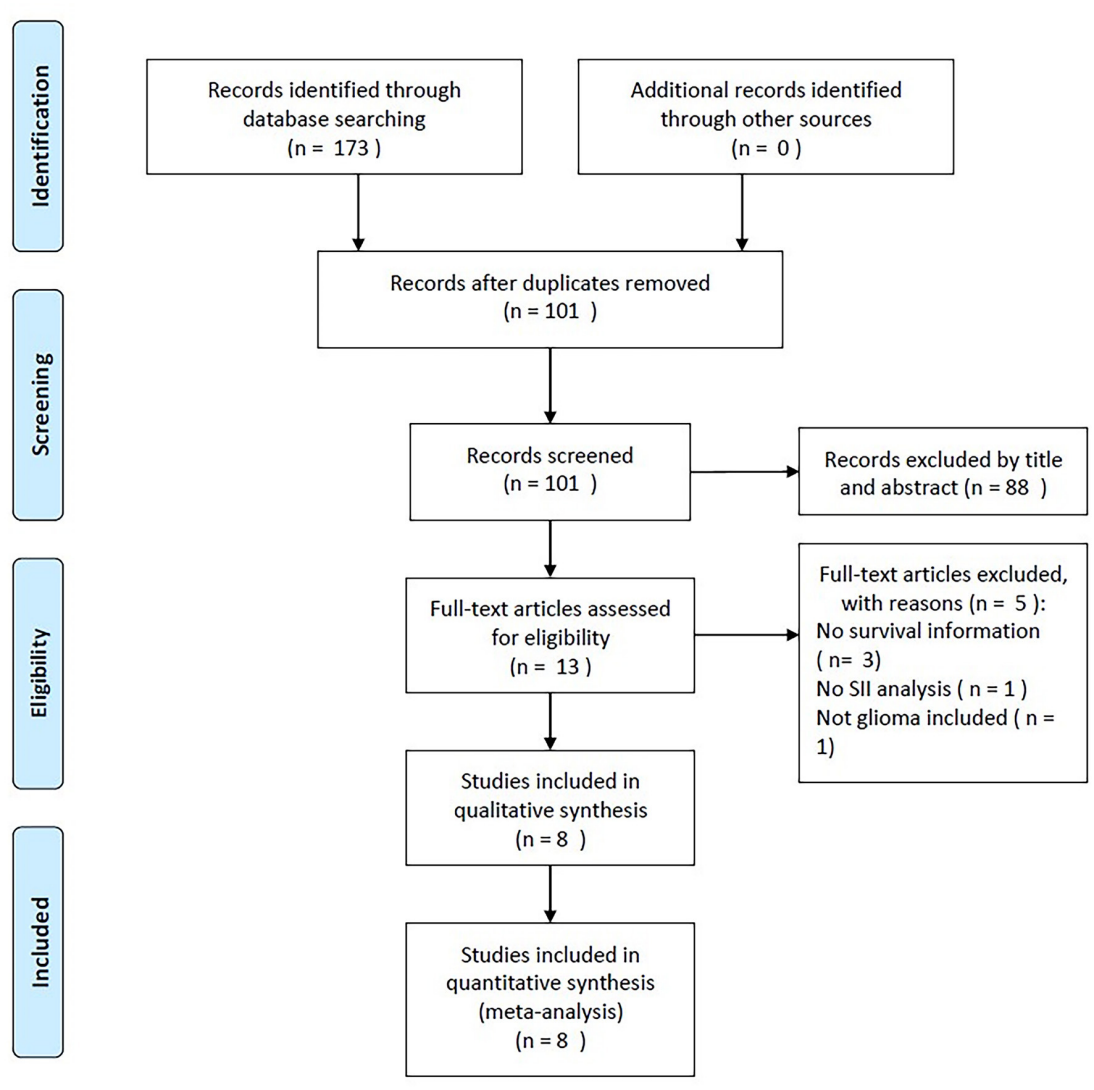
Flowchart of literature retrieval.

Initially, 173 records were identified, but only 101 studies remained after duplicate records were removed (Figure 1). Based on the title and abstract of the studies, 88 studies were excluded as they were irrelevant studies on patients without glioma or animal studies, and the full texts of 13 studies were evaluated further. In addition, five studies were discarded based on the reasons such as the nonavailability of survival information (*n* = 3), no analysis of the SII (*n* = 1), and the enrollment of overlapped patients (*n* = 1). As a result of this meta-analysis, we enrolled eight articles including 1,426 cases (15–22) (Figure 1).

### Included studies' basic characteristics

Table 1 displays baseline study features. To be specific, articles enrolled in this meta-analysis were published between 2019 and 2022 and were all in English (15–22). A total of four studies were performed in China (15, 16, 19, 22), two studies were carried out in Turkey (17, 18), and one each in Italy (21) and Poland (20), respectively. A sample size of 77 to 358 was obtained, with 168 representing the median. All studies were of retrospective design. A total of six studies included patients with GBM (16–18, 20–22), one study recruited patients with glioma grade III–IV (15), and one study enrolled patients with medulloblastoma (MB) (19). The SII was characterized by a median value of 914.5, with the cut-off value ranging from 324.38 to 2,300. For the following subgroup analysis, we selected the cut-off value of 1,000 for the SII because this value is close to the median value and divides the included data into two equal groups (4:4). A receiver operating characteristic (ROC) curve analysis was conducted to determine the threshold of seven articles (16–22), and there was one article (15) using X-tile software. All eight studies analyzed the prognostic role of the SII for OS (15–22), and three studies reported the association between SII and PFS (17, 18, 22). The HRs and 95% CIs were provided by four studies using multivariate analysis (15, 17, 18, 22), and four studies applied univariate analysis (16, 19–21). There was a range of NOS scores from 6 to 8, suggesting that our enrolled articles were of high quality.

**Table 1:** Basic characteristics of included studies in this meta-analysis.

**References**	**Country**	**Sample size**	**Age (year) median (range)**	**Gender (M/F)**	**Study period**	**Pathology**	**Treatment**	**Follow–up (month) median (range)**	**Cut–off value**	**Cut–off determination**	**Survival outcomes**	**Survival analysis**	**NOS score**
Liang et al. (15)	China	169	53 (21–91)	99/70	2014–2016	Glioma	Surgery	1–31	324.38	X–tile	OS	Multivariate	7
Lv et al. (16)	China	192	55	113/79	2006–2018	GBM	Surgery	57.5 (2–151)	718	ROC curve	OS	Univariate	7
Topkan et al., (17)	Turkey	167	57 (26–80)	110/57	2007–2017	GBM	Mixed	13.8 (1.1–108.3)	565	ROC curve	OS, PFS	Multivariate	8
Yilmaz et al. (18)	Turkey	120	60 (20–81)	72/48	2010–2020	GBM	Mixed	17 (1–67)	1,111	ROC curve	OS, PFS	Multivariate	8
Zhu et al. (19)	China	111	10 (1–48)	71/40	2001–2021	MB	Surgery	To Apr 2021	2,278	ROC curve	OS	Univariate	7
Jarmuzek et al. (20)	Poland	358	62.3 (21.9–84.7)	195/163	2004–2021	GBM	Mixed	7 (1–120)	2,300	ROC curve	OS	Univariate	6
Pasqualettiet al. (21)	Italy	77	64 (26–84)	43/34	2010–2020	GBM	Radio-Chemotherapy	23 (3–94)	1,200	ROC curve	OS	Univariate	7
Shi et al. (22)	China	232	< 65 years: 193; ≥65 years: 39	105/127	2014–2018	GBM	Mixed	1–70	659.1	ROC curve	OS, PFS	Multivariate	8

M, male; F, female; GBM, glioblastoma; MB, medulloblastoma; OS, overall survival; PFS, progression-free survival; ROC, receiver operating characteristic.

NOS, Newcastle–Ottawa Scale.

### SII and OS in glioma

**Figure 2:**
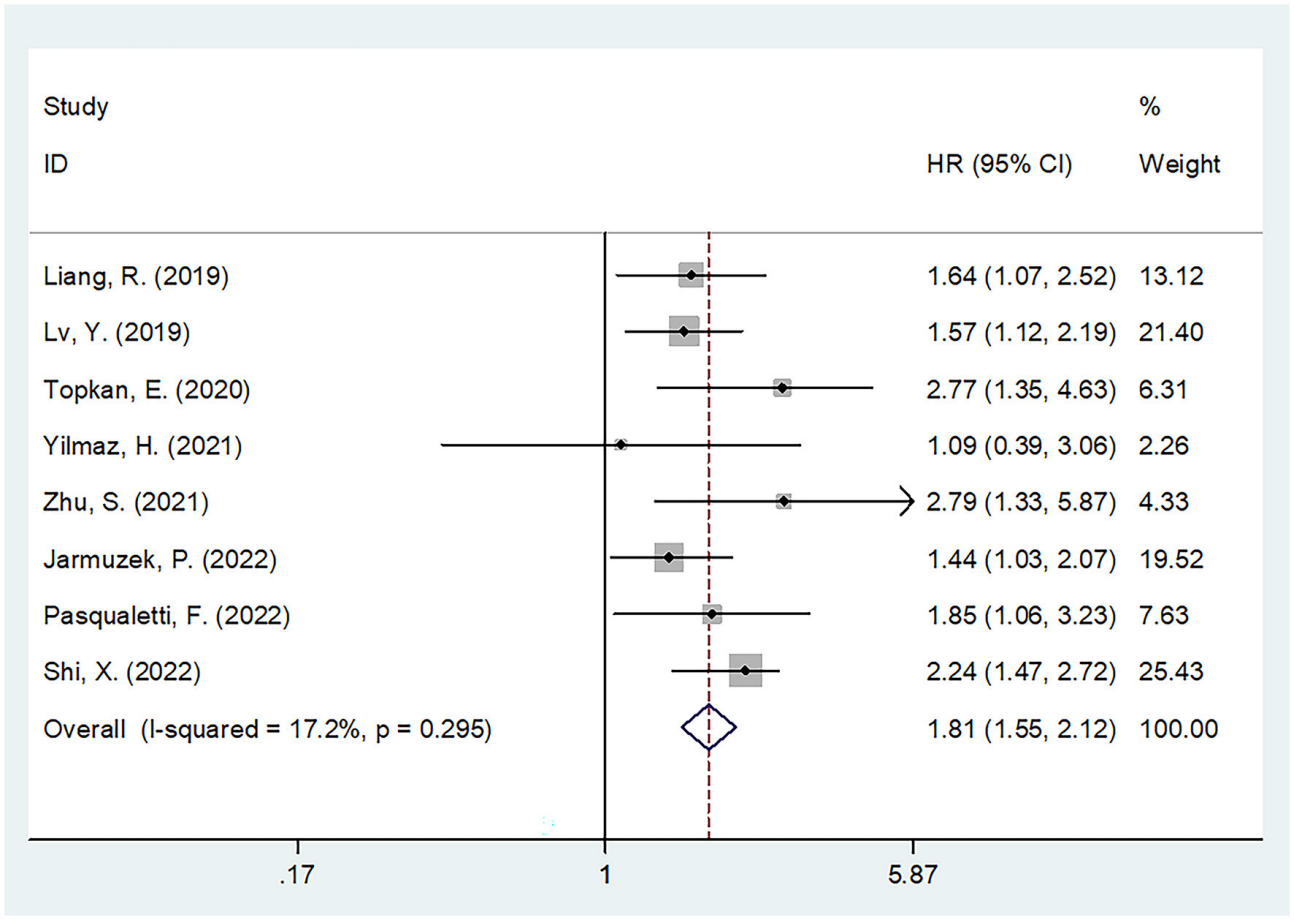
Forest plot of the association between SII and overall survival of glioma.

In each of the eight studies involving 1,426 patients, the SII was assessed for its prognostic significance of overall survival in patients with glioma (15–22). Because of insignificant heterogeneity (I^2^ = 17.2%, Ph = 0.295), this study adopted the fixed-effects model. The pooled results included HR = 1.81, 95% CI = 1.55–2.12, and a *p* < 0.001, indicating that the high SII level significantly predicted poor OS of glioma cases (Figure 2; Table 2). Furthermore, subgroup analyses were carried out based on the region, threshold level, treatment, and type of survival analysis. According to the subgroup analysis shown in Table 2, a high SII value has an effect on OS in all different subgroups of glioma cases (*p* < 0.05).

**Table 2:** Subgroup analysis of the prognostic value of SII for OS and PFS in patients with glioma.

**Subgroups**	**No. of studies**	**No. of patients**	**Effects model**	**HR (95%CI)**	***p***	**Heterogeneity I** ^**2**^ **(%) Ph**	
**Overall survival**							
Total	8	1,426	Fixed	1.81 (1.55–2.12)	< 0.001	17.2	0.295
**Region**							
Asia	6	991	Fixed	1.92 (1.61–2.31)	< 0.001	21.4	0.273
Non-Asia	2	435	Fixed	1.54 (1.15–2.08)	0.004	0	0.459
**Pathology**							
GBM	6	1,146	Fixed	1.80 (1.52–2.14)	< 0.001	27.9	0.225
Glioma/MB	2	280	Fixed	1.87 (1.29–2.71)	0.001	32.3	0.224
**Treatment**							
Surgery	3	472	Fixed	1.70 (1.33–2.18)	< 0.001	0	0.376
Mixed	4	877	Random	1.88 (1.34–2.64)	< 0.001	50.5	0.109
Radio-Chemotherapy	1	77	-	1.85 (1.06–3.23)	0.031	-	-
**Cut-off value**							
< 1,000	4	760	Fixed	1.92 (1.58–2.32)	< 0.001	29.1	0.237
≥1,000	4	666	Fixed	1.63 (1.25–2.12)	< 0.001	8.2	0.352
**Survival analysis**							
Multivariate	4	688	Fixed	2.04 (1.63–2.56)	< 0.001	19.1	0.294
Univariate	4	738	Fixed	1.63 (1.32–2.02)	< 0.001	0	0.434
**Progression-free survival**							
Total	3	519	Fixed	1.87 (1.44–2.43)	< 0.001	43.8	0.169
**Cut-off value**							
< 1,000	2	399	Fixed	2.01 (1.53–2.65)	< 0.001	0	0.921
≥1,000	1	120	-	0.80 (0.32–2.01)	0.632	-	-

GBM, glioblastoma; MB, medulloblastoma; OS, overall survival; PFS, progression-free survival.

### SII and PFS in glioma

**Figure 3:**
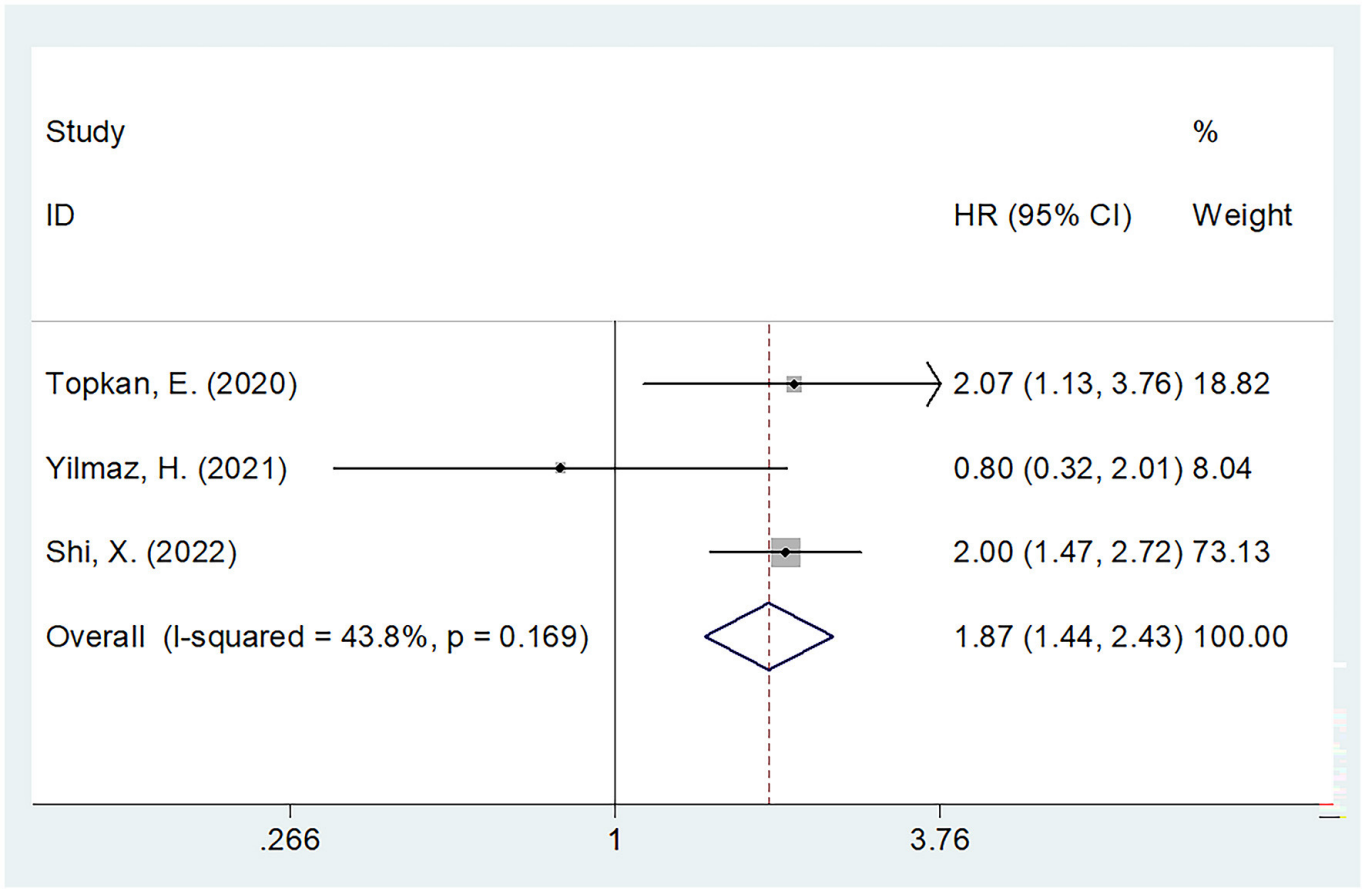
Forest plot of the association between the SII and progression-free survival of glioma.

Association between SII with PFS in glioma was presented in three studies with 519 patients (17, 18, 22). Due to the presence of insignificant heterogeneity (I^2^ = 43.8%, Ph = 0.169), this study utilized the fixed-effects model (Table 2). As suggested by the combined data, an increased SII level predicted dismal PFS (HR = 1.87, 95% CI = 1.44–2.43, *p* < 0.001) in patients undergoing glioma (Figure 3, Table 2). Due to the limited sample size, the subgroup analysis was carried out by a cut-off value. Based on the subgroup analysis, an increased SII level significantly predicted PFS when the cut-off value of GNRI <1,000 (HR = 2.01, 95% CI = 1.53–2.65, *p* < 0.001) (Table 2).

### The relationship between the SII and clinicopathological factors in glioma

The relationship between SII and clinicopathological factors of patients with glioma was reported in five studies including 871 patients (15–17, 19, 22). These clinicopathological characteristics were as follows: gender (male vs. female), Karnofsky performance score (KPS) (<80 vs. ≥80), symptom duration (≥3 vs. < 3 months), and Ki-67 (≥30 vs. < 30%). Based on the combined results, an increased SII level was remarkably related to Ki-67 ≥30% (OR = 1.72, 95%CI = 1.10–2.69, *p* = 0.017) (Table 3). Nonetheless, SII was not related to sex (OR = 1.05, 95%CI = 0.78–1.41, *p* = 0.734), KPS (OR = 0.64, 95%CI = 0.17–2.37, *p* = 0.505), or symptom duration (OR = 1.22, 95%CI = 0.37–4.06, *p* = 0.745) (Table 3).

**Table 3:** Association between SII and clinicopathological features in patients with glioma.

**Factors**	**No. of studies**	**No. of patients**	**Effects model**	**OR (95%CI)**	***p***	**Heterogeneity I** ^**2**^ **(%) Ph**	
Gender (male vs. female)	5	871	Fixed	1.05 (0.78–1.41)	0.734	0	0.845
KPS (< 80 vs. ≥80)	3	510	Random	0.64 (0.17–2.37)	0.505	85.0	0.001
Symptom duration (≥3 months vs. < 3 months)	2	278	Random	1.22 (0.37–4.06)	0.745	74.3	0.049
Ki-67 (≥30 vs. < 30%)	2	424	Fixed	1.72 (1.10–2.69)	0.017	0	0.888

KPS, Karnofsky performance score.

### Sensitivity analysis

**Figure 4:**
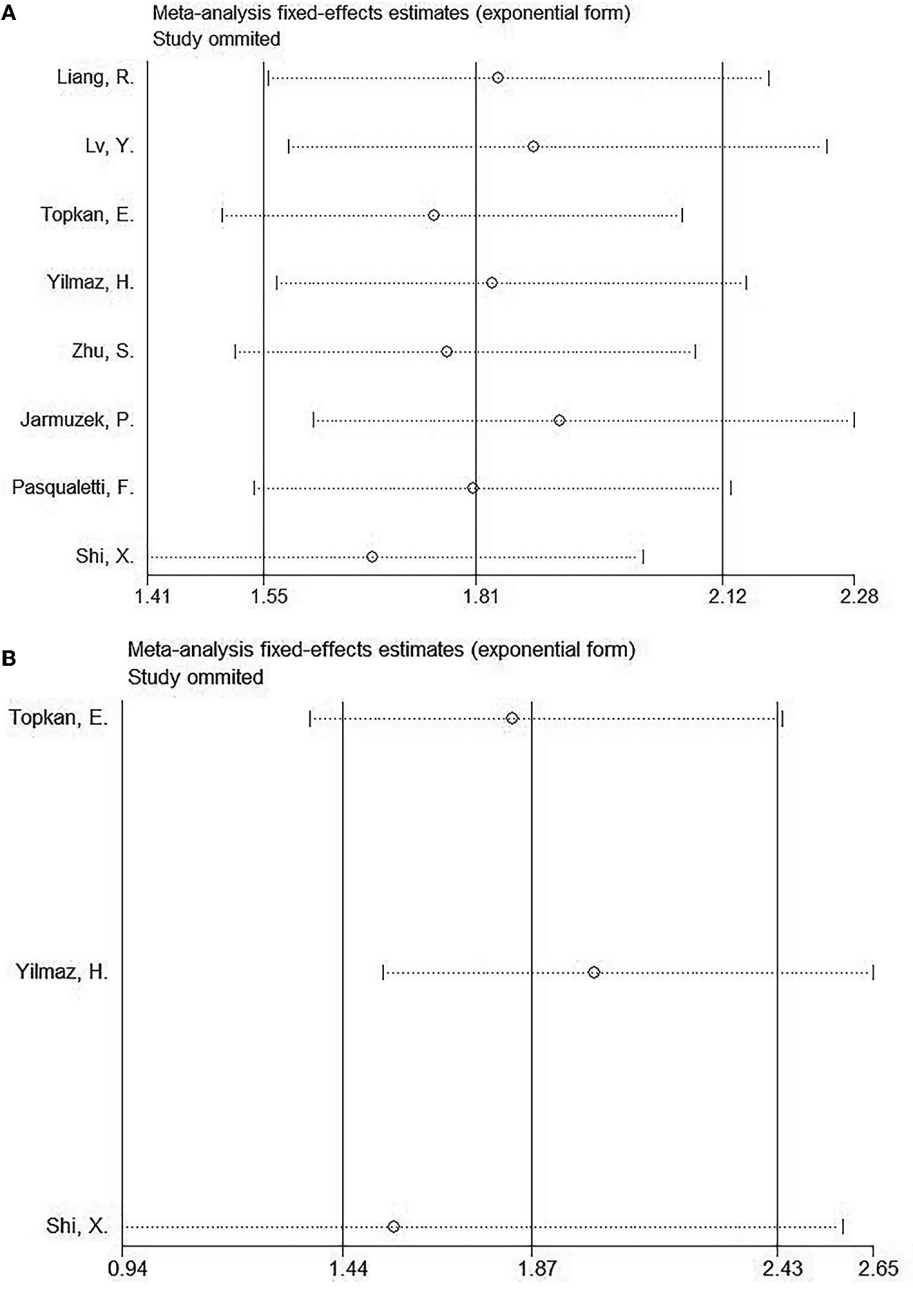
Sensitive analyses of included studies in this meta-analysis. **(A)** OS and **(B)** PFS.

In order to identify the source of heterogeneity and to document the stability of our results, we performed a sensitivity analysis by omitting one study at a time. As shown in Figure 4, following the elimination of the included studies, the pooled HRs did not significantly change, indicating that the results of our study were stable.

### Publication bias

**Figure 5:**
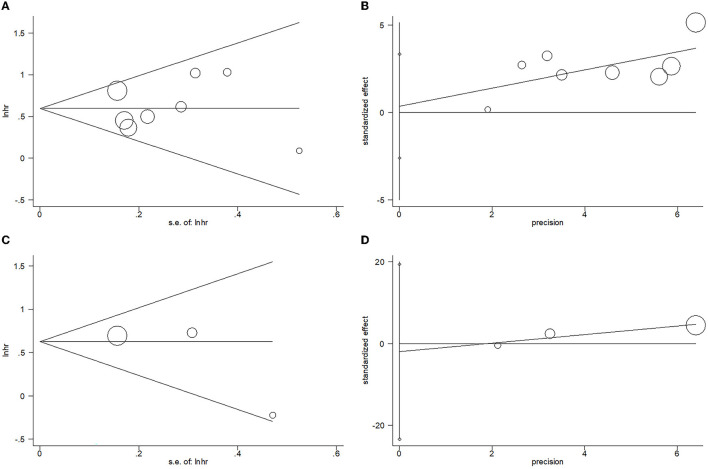
Results of the analysis of publication bias. **(A)** OS, Begg's test, *p* = 1; **(B)** OS, Egger's test, *p* = 0.776; **(C)** PFS, Begg's test, *p* = 0.296; and **(D)** PFS, Egger's test, *p* = 0.448.

Egger's test and Begg's test were adopted to analyze publication bias in the present meta-analysis. For OS, the *p*-value of Begg's test is 1, and the *p*-value is 0.776 for Egger's test. For PFS, the results included a *p*-value of 0.296 for Begg's test and a *p*-value of 0.448 for Egger's test. As shown in Figure 5, the funnel plots are asymmetric, and obvious publication bias was detected for OS or PFS.

## Discussion

In previous studies, the SII was explored as the factor to predict the patient prognosis; however, the results were conflicting (15–22). In order to accurately clarify this issue, the data from eight studies comprising 1,426 patients were pooled in this meta-analysis. According to our results, both OS and PFS of patients with glioma were significantly affected by an elevated SII value. Subgroup analyses verified that the SII could be used in predicting the prognosis of glioma. Furthermore, we also identified a positive relationship between SII and Ki-67 ≥30% in patients with glioma. Overall, this meta-analysis indicated the significant relation between SII and OS/PFS of glioma cases. Patients with glioma with high SII may suffer from aggressive cancer of high proliferation. As far as we know, the present study is the first to explore the prognostic and clinicopathological value of SII in glioma.

Increasing evidence suggests that inflammation and immune responses may promote tumor progression by creating conditions that facilitate metastasis within the tumor microenvironment (24, 25). Hematological parameters based on immune cells are easily available for survival prognostications in patients with cancer (26, 27). The SII is calculated by platelet count × neutrophil count/lymphocyte count. Based on the earlier equation, an increased platelet count, an increased neutrophil count, and/or a decreased lymphocyte count could result in an elevated SII. Although the precise mechanisms of SII's prognostic role in glioma are unclear, they can be explained by the following. First, platelets can protect tumor cells from cytolysis and can promote metastasis (28). In addition, platelets can promote tumor progression through platelet factor and vascular endothelial growth factor (VEGF), together with platelet-derived growth factor (PDGF) (29). Platelets can also secrete inflammatory cytokines such as interleukin (IL)-6 and TNF-α to facilitate tumor cell metastases (30). Second, neutrophils have significant tumor-promoting activity. Neutrophils can induce myeloid-derived suppressor cells (MDSCs), and MDSCs are myeloid cells that produce reactive oxygen species and arginase to suppress T lymphocyte activation. Therefore, neutrophils can educate an immunosuppressive activity in the tumor microenvironment. Third, lymphocytes, especially tumor-infiltrating T cells, have an important effect on T- cell-dependent anticancer response. Tumor-infiltrating lymphocytes (TILs) can secrete IL-4, IL-5, and the tumor necrosis factor to regulate the angiogenesis, proliferation, apoptosis, and metastasis of tumor cells. In addition, lymphocyte infiltration in tumors is related to the superior prognosis of patients with cancer (31). Moreover, in a recent quantitative digital analysis, the researchers reported that high expressions of CD8+ and CD163+ cells were significantly connected with inferior survival in patients with GBM (32). According to their observations, a direct correlation was found between CD163 values and CD8 or PDL1/PD1 infiltration in patients with GBM (32). Therefore, the high SII value can be adopted for predicting the dismal prognosis of glioma cases. As reported in this meta-analysis, a high SII value was significantly associated with poor OS and PFS in patients with glioma. Therefore, in clinical settings, patients with glioma with a pretreatment SII value of >1000 may have a high risk of poor survival and disease progression. Notably, the results of included studies differed, which may be caused by selection bias because the eligible studies enrolled patients by different selection criteria (15–18).

The SII is widely suggested to have an essential function in different cancer types by meta-analyses in recent years (33–36). As suggested by Li et al., an increased SII level before treatment predicted low survival in HCC cases after transcatheter arterial chemoembolization in the meta-analysis enrolling 3,557 cases (35). Huang et al. showed with cases of non-small cell lung cancer (NSCLC) who showed an increased SII level had a poor OS and a poor PFS based on their meta-analysis of 17 enrolled studies (36); moreover, they had a higher pathological stage (II–III) relative to patients with normal SII levels (36). According to a recent meta-analysis involving 2,642 patients, breast cancer (BC) cases who had increased SII levels were associated with poor OS, recurrence-free survival (RFS)/disease-free survival (DFS), and dismal distant metastasis-free survival (DMFS) than those with a low SII (37). Head and neck cancer cases who had an increased SII level before treatment were associated with poor OS, DFS, and patient-reported survival in a meta-analysis of 12 studies (33). As a result of a meta-analysis of 1,402 patients with cholangiocarcinoma undergoing invasive surgery, Liu et al. found that an increased SII level independently predicted dismal OS (34). Our findings in the current meta-analysis conformed to SII's role in predicting other cancer prognoses (33–36).

As a meta-analysis, certain limitations should be noted in the present study. First, the enrolled articles were retrospective, which could cause inherent heterogeneity. Second, the total number of studies included was relatively small. There were eight studies involved in this meta-analysis, and only 1,426 patients were recruited. Therefore, selection bias may exist. Third, study cut-off values for the SII were not uniform, which may have affected the combined HRs and 95% CIs. For subgroup analysis, we selected a cut-off value of SII = 1,000 because this value is close to the median value and divides the included data into two equal groups (4:4). The subgroup analysis indicated that high SII was significantly associated with worse OS irrespective of SII cut-off values (Table 2). In addition, an elevated SII value was correlated with poor PFS when the cut-off value is <1,000 (Table 2). Therefore, further prospective studies with a large sample size should be conducted for validating our meta-analysis results.

## Conclusion

The overall conclusion of the present meta-analysis was that an increased SII level was related to worse OS and PFS after glioma. Moreover, patients with glioma with high SII have a positive relationship with a Ki-67 of ≥30%.

## Data availability statement

The original contributions presented in the study are included in the article/supplementary material, further inquiries can be directed to the corresponding author.

## Author contributions

All authors listed have made a substantial, direct, and intellectual contribution to the article and approved it for publication.
